# Protection of N‑Type (Ni,Fe)TiSb Half-Heusler Materials against Static and Cyclic Oxidation Using a Si-Doped Cr Coating

**DOI:** 10.1021/acsomega.5c09137

**Published:** 2025-10-17

**Authors:** Mikdat Gurtaran, Zhenxue Zhang, Xiaoying Li, Hanshan Dong

**Affiliations:** † Faculty of Engineering, Material Science and Engineering, Izmir Institute of Technology, 35430 Izmir, Turkey; ‡ Interdisciplinary Research Centre for Advanced Materials, King Fahd University of Petroleum and Minerals, 31261 Dhahran, KSA; § School of Metallurgy and Materials, 1724 The University of Birmingham, B15 2TT Birmingham, U.K.

## Abstract

In this study, Cr–Si coatings were deposited on N-type (Ni,Fe)­TiSb thermoelectric (TE) materials by using a closed-field unbalanced magnetron sputtering PVD technique. Oxidation behavior was evaluated under both isothermal (static) conditions (500 °C for 10 h and 600 °C for 50 h) and thermal cycling regimens (500 and 600 °C for 10 or 50 1 h cycles). Mass gain, surface morphology, cross-sectional microstructure, elemental distribution, and phase composition were examined by using scanning electron microscopy (SEM), energy-dispersive X-ray spectroscopy (EDX), and X-ray diffraction (XRD). Regardless of exposure mode, uncoated samples oxidized severely: a duplex scale formed, consisting of an outer TiO_2_ layer and a subjacent NiSb-rich zone, accompanied by extensive cracking and delamination. In sharp contrast, the Cr–Si coatings remained thermally stable and highly oxidation-resistant, maintaining the substrate’s integrity during both static and cyclic tests. After exposure, coated samples showed negligible mass gain, no discernible morphological change, and no mechanical damage, confirming that the Cr–Si layer markedly enhances thermal durability and prevents surface degradation.

## Graphical abstract

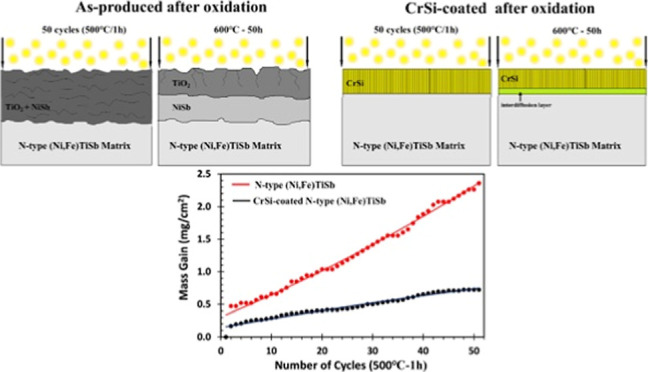

## 1 Introduction

The continuous increase in global energy demand has made waste energy recovery an increasingly critical area of focus.^1^ Moreover, growing concerns over the environmental impact of fossil fuels have prioritized the development of eco-friendly technologies capable of delivering a clean energy solution.^2,3^ Among these, thermoelectric (TE) technology, which converts waste heat into clean electricity, has gained significant momentum over the past two decades.^4^ Central to the advancement of efficient TE modules is the synthesis of TE materials with high dimensionless figures of merit (zT), which reflect the thermoelectric efficiency by integrating the effects of electrical conductivity, thermal conductivity, the Seebeck coefficient, and the operating temperature of the module.^5–7^

Among TE materials, half-Heusler (HH) compounds have become attractive in recent years due to their high efficiency (zT) at medium-to-high temperatures.^8–10^ Notably, zT values in the range of 1.05–1.5 have been reported at elevated temperatures between 825 and 1000 K.^11–14^ However, the practical deployment of these materials in long-service-life TE modules remains challenging due to their insufficient physical and chemical stability under high-temperature conditions. In particular, surface oxidation^15–18^ and sublimation—especially in HH materials containing Sn and/or Sb^19^—can alter the material’s composition, potentially degrading its TE performance and mechanical integrity. For example, Gu et al.^20^ investigated the formation of three distinct oxide layers on the surface of the ZrCoSb-based HH materials after oxidation at 873–1073 K. Similarly, Appel et al.^16^ studied the oxidation behavior of ZrNiSn-based HH materials and found that ZrO_2_ and SnO_2_ dominated the oxidized surface at approximately 1000 K. In the previous study, we systematically investigated the oxidation characteristics of the N-type (Zr,Ti)­Ni­(Sn,Sb) and P-type (Zr,Ti)­Co­(Sn,Sb) TE materials. The results showed significant changes in surface composition after cyclic oxidation at 873 K, which caused surface spallation and cracking, ultimately compromising the structural integrity of the materials.^21^

Given the above-mentioned challenges, it is evident that either the oxidation resistance of HH materials must be intrinsically improved^22^ or these materials must be externally protected from oxidation.^23^ However, the first option is inherently limited, as it is challenging to simultaneously achieve a high thermoelectric efficiency and strong oxidation resistance. For instance, dopants such as Sn and Sb can significantly enhance thermoelectric performance; however, they are susceptible to sublimation even at low temperatures. Likewise, elements like Ni, Co, Ti, and Zr, despite their beneficial roles in TE performance, are prone to oxidation at high temperatures when incorporated into HH alloys. Therefore, to preserve both structural integrity and TE efficiency, it becomes essential to protect HH materials by employing advanced surface engineering strategies, such as the application of novel, thermally stable protective coatings.

Several approaches have been explored in the literature to protect TE materials from oxidation, including the use of glass coatings,^24,25^ enamel,^26^ SiC,^27^ and hybrid coatings.^28^ Additionally, a surface peroxidation strategy has been proposed to enhance oxidation resistance,^20^ promoting the preferential formation of a thin superficial oxide layer to inhibit inward oxygen diffusion. While this surface passivation can be effective, it does not guarantee long-term stability of the oxide layer under extended high-temperature operation. To address this limitation, we previously developed thermally stable Cr–Si-based coatings, which were successfully deposited on N-type (Zr,Ti)­Ni­(Sn,Sb) and P-type (Zr,Ti)­Co­(Sn,Sb) HH materials. The promising results, published in ref,^29^ demonstrated the potential of Cr–Si coatings to protect HH alloys against high-temperature oxidation, laying the groundwork for further research in this area. Building on this previous experience, the current study focused on the application of Cr–Si coatings to (Ni,Fe)­TiSb TE materials, deposited using a multitarget closed-field unbalanced magnetron sputtering PVD system. The coated samples were subjected to both static and cyclic oxidation to assess their high-temperature oxidation resistance. Mass gain, surface morphology, microstructure, and phase evolution were systematically analyzed to evaluate the protective performance of the Cr–Si coatings. This study provides new insights into enhancing the thermal durability of HH TE materials and offers a promising route for protecting other TE systems operating in harsh environments.

## 2 Experimental Section

### 2.1 Materials and Sample Preparation

An N-type (Ni,Fe)­TiNi thermoelectric material was produced by mechanically alloying industrial-grade powders of Ni, Fe, Ti, and Ni in specific proportions, as outlined in Table 1. MBN Nanomaterialia (Treviso, Italy) developed a special high-energy ball milling technique to transfer kinetic energy from the milling balls to the powder particles, resulting in powders with high surface activity and minimal contamination. This process was carried out under an argon atmosphere to avoid oxidation. After that, the milled powders were transferred into a graphite die (20 mm inner diameter, 15 mm thickness) inside an argon-filled glovebox to prevent contamination and possible oxidation. The die was placed in an HPD-25 spark plasma sintering (SPS) system (FCT Systeme GmbH) with graphite punches. Sintering was carried out at 900 °C for 4 min under a uniaxial pressure of 500 kg/cm^2^, with a heating rate of 100 °C/min in an argon environment. The produced N-type TE legs were cut into 3 × 3 × 3 mm^3^ blocks and ground using SiC abrasive papers up to 1200 grit. The sample surfaces were subsequently cleaned in an acetone bath to remove any contaminants and then dried at room temperature.

**Table 1:** Chemical Composition of the N-Type (Ni,Fe)­TiSb Thermoelectric Material

sample code	chemical formula	composition (at. %)			
N	(Ni_0.7_Fe_0.3_)_0.33_Ti_0.33_Sb_0.33_	Ni	Fe	Ti	Sb
		18.1	7.3	21.1	53.5

### 2.2 Deposition of Cr–Si Coatings

Cr–Si coatings were deposited onto N-type (Ni,Fe)­TiSb thermoelectric materials using an unbalanced closed-field ion-plating system. Magnetron sputtering physical vapor deposition (PVD) equipment enables separate Cr and Si cathode targets, allowing an easy adjustment of the Cr/Si ratio by varying the target currents. The coating process consists of three main steps: beginning with ion cleaning of the sample surfaces using low currents of 0.05 A for Si and 0.2 A for Cr for 15 min, under a relatively high bias voltage of 200 V. Subsequently, the target currents were increased to their final values (2 A for Cr and 0.3 A for Si), while the bias voltage was gradually reduced from 200 to 40 V over a period of 5 min. In the final stage, Cr–Si coatings were deposited for 1 h in an argon atmosphere at a pressure of 13.33 × 10^–2^ Pa. During this period, the samples were rotated at 5 rpm to ensure a uniform coating deposition.

### 2.3 Static and Cyclic Oxidation Testing

To perform the cyclic oxidation testing, the samples were placed in chemically stable ceramic containers to prevent any reaction or contamination during the insertion and removal of samples from the furnace. Cyclic oxidation was performed in an open-air muffle furnace. The samples were quickly loaded into the furnace at 350 °C. Afterward, the temperature was then increased to 500 or 600 °C within 2 or 5 min, respectively, where the samples were kept for 1 h for oxidation. Following this, the temperature was lowered back to 350 °C by opening the furnace door, and the samples were removed and allowed to cool slowly to room temperature. This procedure constituted one oxidation cycle and was repeated 10 or 50 times for each sample. After every cycle, the samples were weighed using a high-precision Ohaus scale with a sensitivity of 10^–4^ g. The surface area of each sample was calculated individually, and the specific mass gains were measured accordingly.

The static oxidation tests were conducted using the same procedure as the cyclic oxidation tests; the samples were inserted into and removed from the furnace at 350 °C. Subsequently, static oxidation was performed at 500 °C for 10 h and at 600 °C for 50 h. Sample codes and corresponding oxidation parameters are listed in Table 2.

**Table 2:** Sample Codes and Their Corresponding Processing Parameters

oxidation method	temperature (°C)	duration (hours)	sample codes	
			uncoated samples	Cr–Si-coated samples
			N	CN
static	500	10 h	N5	CN5
	600	50 h	N6	CN6
cyclic	500	1 h × 10 cycles	N5–10	CN5–10
		1 h × 50 cycles	N5–50	CN5–50
	600	1 h × 10 cycles	N6–10	CN6–10

### 2.4 General Characterization

Surface morphology of both the as-produced and Cr–Si-coated (Ni,Fe)­TiSb thermoelectric materials was examined before and after static and cyclic oxidation using scanning electron microscopy (SEM, Jeol 7000) in both secondary electron imaging (SEI) and backscattered electron imaging (BEI) modes. To assess the chemical composition and elemental distribution within the coating and at the interface between the coating and substrate after oxidation tests, energy-dispersive X-ray spectroscopy (EDX), integrated with SEM, was used. The elemental distribution in the coating layers and substrate material was also characterized by EDX mapping. Surface phase analysis was carried out using a ProtoA X-ray diffractometer with a Cu Kα radiation source (λ = 1.540598 Å), operating in coupled scan mode at a fixed resolution of 0.01493° in 2θ. Measurements were taken over a 2θ range of 20°–90°, with a step size of 0.5° and a dwell time of 2 s per step. The resulting XRD patterns were identified by using Highscore Plus software.

## 3 Results

### 3.1 Microstructure of Cr–Si Coating Layers

The surface morphology of the Cr–Si coatings on the (Ni,Fe)­TiSb thermoelectric material and the cross-sectional structure on the Si wafer are shown in Figure 1. The surface morphology of the Cr–Si coating reveals that the grains are tightly packed, resulting in a highly dense and uniform layer with a polycrystalline structure (Figure 1a). EDX analysis indicates that the coating’s chemical composition is approximately 93.76 at. % Cr and 6.24 at. % Si averaged from five different measurement regions, with a standard deviation of ±0.2%. The cross-sectional image shows a fine columnar structure of the coating with no observable pores or spallation, confirming a uniform and dense, compact Cr–Si layer (Figure 1b).

**Figure 1:**
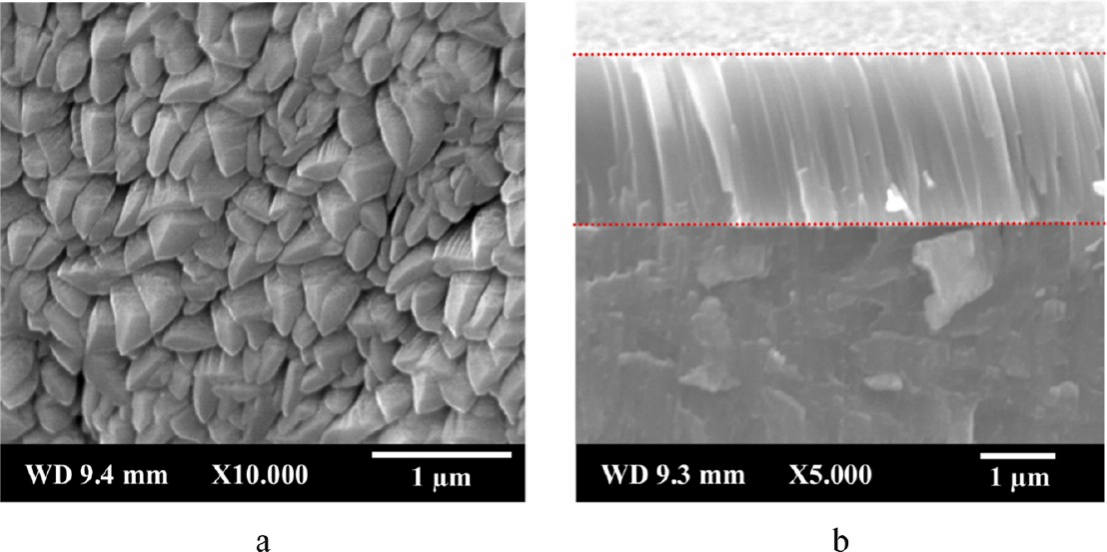
(a) Surface morphology of Cr–Si coatings on the (Ni,Fe)­TiSb TE material and (b) fractured cross-section layer structure of the Cr–Si coating on a Si wafer.

### 3.2 Protection of the N-Type (Ni,Fe)­TiSb TE Material against Static Oxidation

#### 3.2.1 Surface Morphology and Phase Identification

The surface morphologies of statically oxidized N-type (Ni,Fe)­TiSb TE samples N5 and N6 and the Cr–Si-coated sample CN6 are presented in Figure 2. The localized degradation with multiple cracks was observed on the N5 sample (oxidized at 500 °C for 10 h, Figure 2a). EDX analysis on sample N5 (Spectrum 1, Table 3) revealed a high oxygen content of 59.9 at. %, along with significant amounts of Ti (20.3 at. %), suggesting the formation of oxides on the surface. In the N6 sample (600 °C for 50 h, Figure 2b), an observable oxide scale and star-shaped oxide features formed on the surface, accompanied by surface cracks. EDX results from the N6 surface (Spectra 2 and 3, Table 3) showed even higher oxygen levels (73.1–78.3 at. %) and a Ti content, indicating penetration of oxygen into the substrate. The oxide layer appears loosely attached, likely leading to poor long-term stability after 50 h of oxidation. In contrast, the CN6 sample (Figure 2c) shows a uniform surface with no cracks, pores, or oxide-related spallation. EDX analysis (Spectrum 4, Table 3) confirmed the protective nature of the Cr–Si layer, showing low oxygen content (26.7 at. %) and high levels of Cr (67.6 at. %) and Si (5.7 at. %), with no oxide-related products from the thermoelectric substrate material. The fine-grained structure of the Cr–Si coating remains little changed after even longer oxidation testing (at 600 °C for 50 h), indicating excellent high-temperature oxidation resistance.

**Figure 2:**
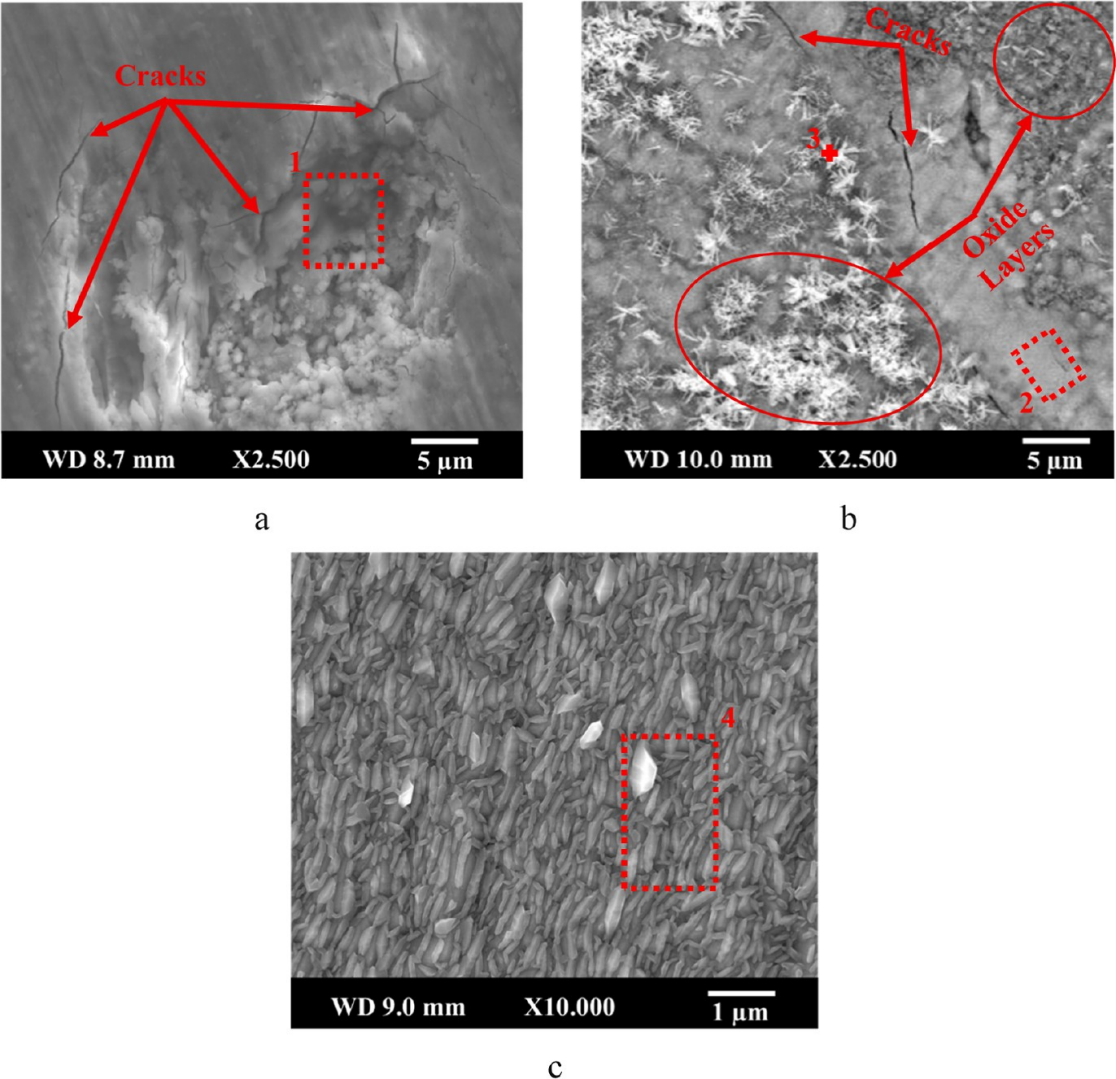
Surface morphology of (a) N5, (b) N6, and (c) CN6 samples.

**Table 3:** EDX Results (at. %) Taken from the Point and Dashed Regions in Figure 2a–c

sample	spectrum	element concentration						
		O	Ni	Ti	Fe	Sb	Cr	Si
N5	1	59.9 ± 0.3	7.2 ± 0.2	20.3 ± 0.2	1.1 ± 0.1	11.5 ± 0.2		
N6	2	73.1 ± 0.3	2.2 ± 0.1	19.1 ± 0.2		5.6 ± 0.1		
	3	78.3 ± 0.3	1.9 ± 0.1	14.0 ± 0.2		5.8 ± 0.1		
CN6	4	26.7 ± 0.2					67.6 ± 0.3	5.7 ± 0.1

The XRD patterns of the as-produced uncoated sample N compared to oxidized samples N5 and N6 are presented in Figure 3a. In the as-produced sample N, the main half-Heusler phase NiTiSb (PDF: 00-052-0904) with the strong crystallographic reflections was identified. In addition, some minor peaks corresponding to the Ti_1.6_Fe_0.7_Sb_1.3_ (PDF: 01-089-6292) compound were detected. After oxidation at 500 °C for 10 h (N5), new diffraction peaks were observed, indicating the formation of several phases corresponding to NiSb (PDF: 03-065-4339), NiSb_2_O_6_ (00-017-0619), and TiO_2_ (PDF: 00-004-0551), and no peaks reflecting the NiTiSb phase were detected, indicating a complete change in the surface phases on the N5 surface. When the oxidation temperature and duration were increased to 600 °C and 50 h (N6), respectively, the phases such as TiO_2_, NiSb_2_O_6_, and NiSb dominated the surface, accompanied by a minor peak corresponding to Ni (PDF: 96-901-2987), which might be due to the decomposition. These phase changes indicate that the surface of the N sample is significantly affected by static oxidation, resulting in substantial differentiation in surface composition from the original. These results are also supported by SEM and EDX analyses (see Figure 2a,b and Table 3), which reveals an increase in oxygen content, leading to a change in surface morphology in the N5 and N6 samples.

**Figure 3:**
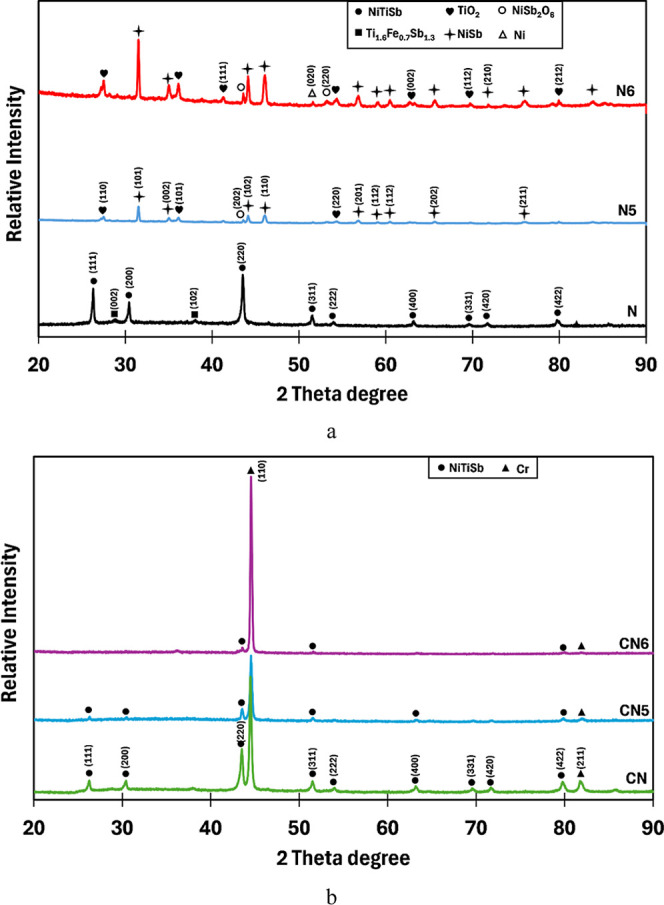
XRD patterns of (a) N, N5, and N6 samples and (b) NC, NC5, and NC6 samples.

In contrast, the XRD patterns for samples CN5 and CN6 remain nearly unchanged after oxidation (500 °C-10 h/600 °C-50 h) compared to the unoxidized Cr–Si-coated N sample (CN), as can be seen in Figure 3b. The main half-Heusler phase of NiTiSb and bcc-structured Cr peaks (PDF: 01-085-1335) were identified on the CN5 and CN6 surfaces, with no oxide-related reflections, like TiO_2_ and NiSb_2_O_6_, or postoxidation products, like NiSb and Ni. This indicates that the Cr–Si coating effectively prevented the oxidation of the thermoelectric material at these temperatures.

#### 3.2.2 Cross-Section SEM Observations and Elemental Distributions

Cross-sectional backscattered electron images of the oxidized uncoated (N5 and N6) and Cr–Si-coated (CN5 and CN6) samples are shown in Figure 4. A distinct grayish top layer and a brighter underlying layer were observed in sample N5 (Figure 4a), indicating significant changes in the surface microstructure due to oxidation. The image also reveals some delamination in the brighter layers, as well as spallation on the top surface, suggesting mechanical degradation of the surface. In contrast, the CN5 sample (Figure 4b) shows an undegraded and nearly the same remaining Cr–Si coating layer after oxidation. The interface between the coating and substrate is sharp and free from delamination, indicating good adhesion and excellent thermal and chemical stability of the Cr–Si coating. In addition, no morphology change, as compared to that of the N5 sample, was observed on sample CN5 after oxidation at 500 °C, suggesting the effective protection of the substrate TE material by the Cr–Si coating against oxidation. No clear chromium or silicon oxide scale is observed on the coating surface as it is either extremely thin or too thin to be detected by SEM and XRD.

**Figure 4:**
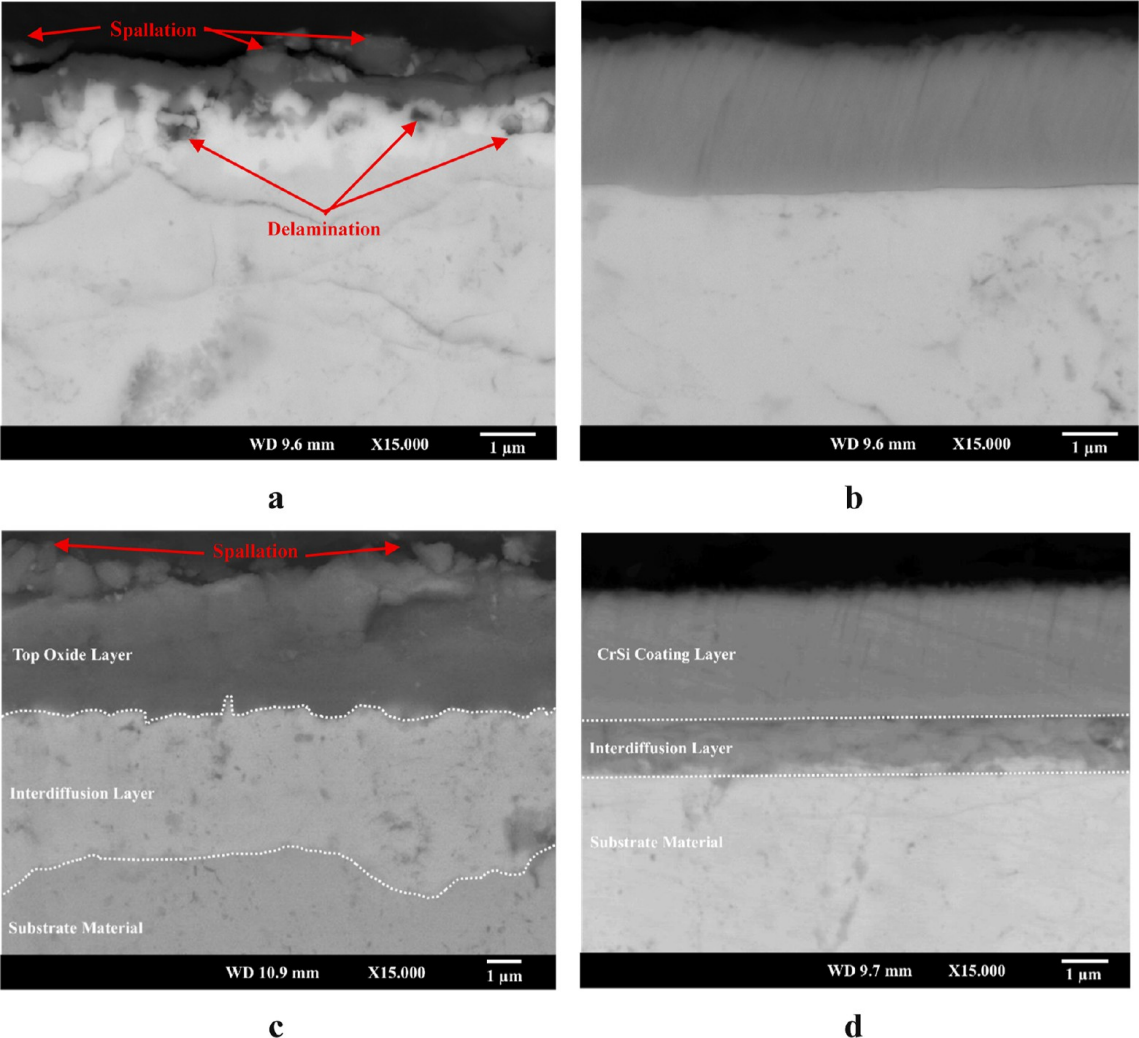
Cross-sectional backscattered electron microscope images of (a) N5, (b) CN5, (c) N6, and (d) CN6 samples.

The N6 sample (Figure 4c), oxidized at 600 °C for 50 h, exhibits a much thicker double-layered structure including a loosely bonded top grayish layer and an underlying brighter layer. This layered structure confirms severe oxidation, which initiates the diffusion of substrate elements, resulting in a dramatic morphological change on the N6 surface and more likely affecting the thermoelectric properties of the material. In the CN6 sample (Figure 4d), the Cr–Si coating remained on the substrate surface with no spallation or cracking even after prolonged exposure at 600 °C. An interdiffusion layer was observed between the Cr–Si coating and the TE material substrate. Nevertheless, excellent structural stability and interface integrity were maintained, confirming the protective function of the Cr–Si coating under severe static oxidation conditions.

The detailed elemental mapping for the N6 and CN6 samples is presented in Figure 5. For sample N6 (Figure 5a), it is evident that the top layer is rich in Ti, as the strong Ti signals are concentrated near the surface. This Ti-rich region is overlapped by oxygen, proving the formation of a TiO_2_ oxide layer on the surface, as already confirmed by XRD analysis (see Figure 3a). Beneath this Ti- and O-rich region, a second layer exhibits high concentrations of Ni and Sb, corresponding to the formation of NiSb. The elemental distribution confirms that the double-layered structure consists of a TiO_2_ oxide scale on top and a NiSb-rich layer beneath, indicating significant phase transformation and oxidation-induced elemental redistribution in the uncoated N6 sample. It should be noted that Fe distribution is uniform in the matrix, and no signals corresponding to Fe were observed in the top Ti-rich and sub-Ni- and Sb-rich layers, possibly due to the low Fe concentration in the TE material, as shown in Table 1.

**Figure 5:**
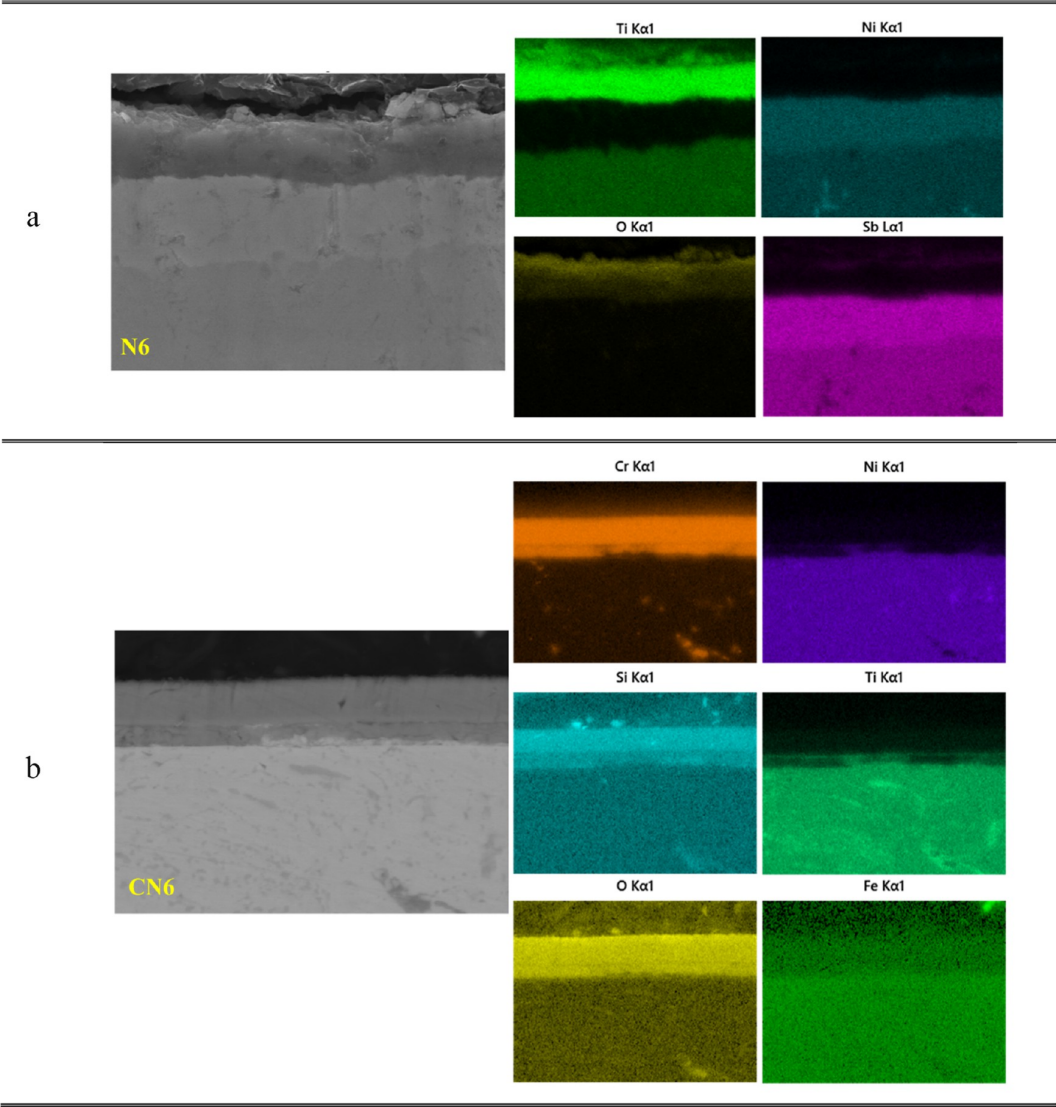
Cross-sectional elemental distribution of (a) N6 and (b) CN6 samples.

As shown in the cross-sectional elemental distribution in Figure 5b, Cr and Si are uniformly concentrated in the upper region within sample CN6, confirming the existence of a Cr–Si coating layer. Beneath the coating, a thin interdiffusion zone can be identified, resulting from the outward diffusion of substrate elements (Ti, Ni, Fe, and Sb) toward the Cr–Si coating layer. Importantly, the oxygen signal is mostly confined to the coating and interdiffusion layers and does not significantly penetrate the thermoelectric material, indicating the effective oxidation barrier function of the Cr–Si coating.

### 3.3 Protection of the N-Type (Ni,Fe)­TiSb TE Material against Cyclic Oxidation

#### 3.3.1 Mass Gain

The mass gain results of cyclically oxidized uncoated (N) and Cr–Si-coated (CN) samples at 500 °C for 50 cycles (N5–50 and CN5–50, Figure 6a) and at 600 °C for 10 cycles (N6–10 and CN6–10, Figure 6b) are presented. For the uncoated N5–50 sample, a continuous mass increase is observed with an increasing number of cycles, reaching approximately 2.3 mg/cm^2^ after the 50th cycle. The polynomial fit equation suggests an accelerating oxidation rate, likely due to the continuous inward diffusion of oxygen during repeated thermal cycling. In contrast, the Cr–Si-coated CN5–50 sample exhibits a significantly lower mass gain, less than 0.5 mg/cm^2^, even after 50 cycles. The near-linear mass gain behavior suggests that while oxygen diffusion into the Cr–Si coating layer still occurs, likely facilitated by its columnar microstructure, it is significantly slowed down due to the dense coating structure, limiting mass gain during cyclic oxidation, especially when compared to the uncoated material.

**Figure 6:**
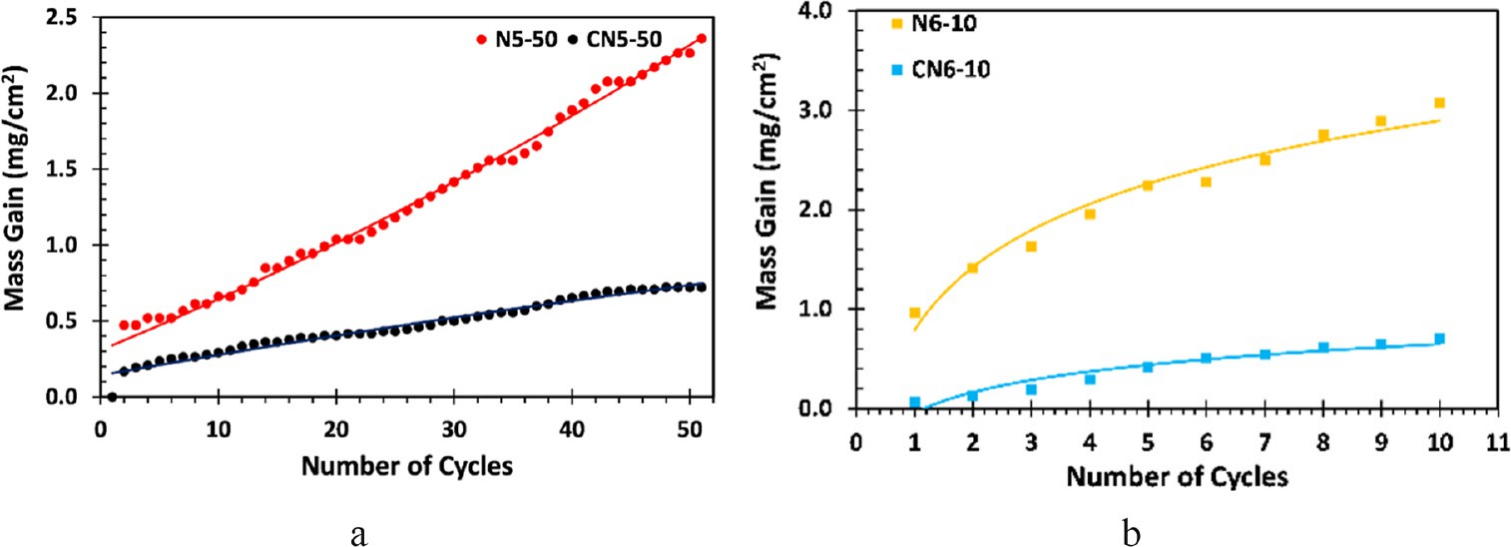
Mass gain comparison of cyclically oxidized N and CN samples at (a) 500 °C for 50 cycles and (b) 600 °C for 10 cycles.

A similar trend is evident for the uncoated N6–10 sample, showing a sharp rise in mass gain over 10 cycles (Figure 6b). However, the mass gain of the CN6–10 sample is much less than that of the N6–10 sample, confirming that the Cr–Si coating layer performs well at higher temperatures.

#### 3.3.2 Surface Morphology and Phase Identification

The surface morphologies of cyclically oxidized uncoated and Cr–Si-coated samples are shown in Figure 7. For the N5–10 sample (Figure 7a), cracks and partial delamination were observed on the surface, meaning poor mechanical stability of the TE material, which is likely caused by repetitive stresses and initiated crack formation during cycling. The EDX analysis of this region (Spectrum 5, Table 4) reveals a high oxygen content (70 at. %) along with significant Ti (15.3 at. %) and some Ni and Sb, more likely indicating the formation of oxide products due to early stage oxidation. The surface damage becomes more severe in the N5–50 sample (Figure 7b), where several polygonal-shaped oxide particles and cracks, along with larger delamination compared to that of N5–10, were observed across the surface, making these materials mechanically unreliable in real TE applications under frequent thermal heating and cooling cycles. Point EDX data (Spectrum 6, Table 4) taken from these oxide particles show high oxygen (77.7 at. %) and titanium (16.0 at. %) contents, confirming titanium oxide formation on the N5–50 surface, while the sharp drop in Ni (1.9 at. %) and Sb (4.3 at. %) content suggests elemental depletion due to oxidation.

**Figure 7:**
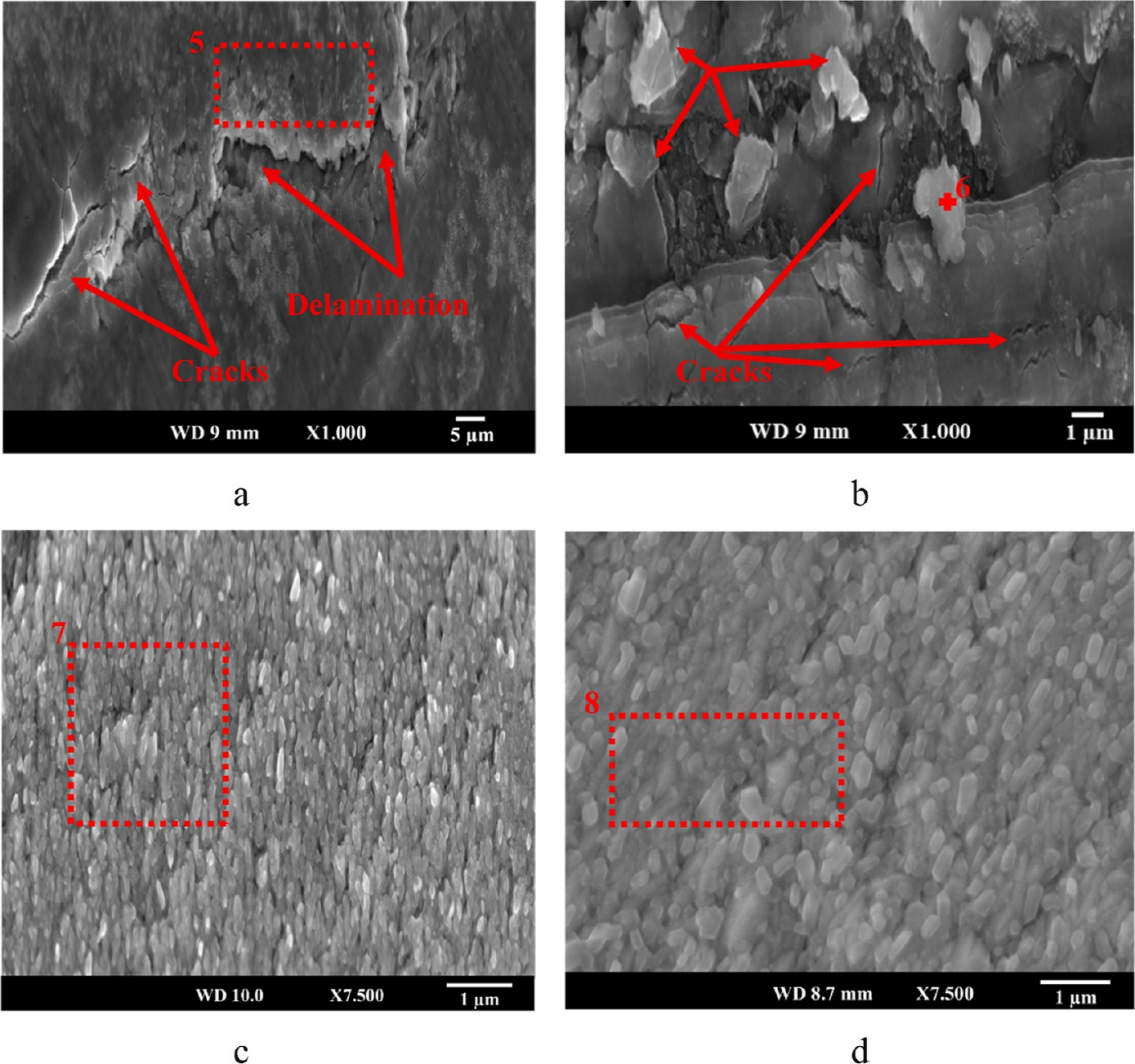
Surface morphology of (a) N5–10, (b) N5–50, (c) CN5–50, and (d) CN6–10 samples.

**Table 4:** EDX Results (at. %) Taken from the Point and Dashed Regions in Figure 7a–d

sample	spectrum	element concentration						
		O	Ni	Ti	Fe	Sb	Cr	Si
N5–10	5	70.0 ± 0.3	4.7 ± 0.1	15.3 ± 0.2	0.2 ± 0.1	9.9 ± 0.2		
N5–50	6	77.7 ± 0.3	1.9 ± 0.1	16.0 ± 0.2	0.1 ± 0.1	4.3 ± 0.1		
CN5–50	7	18.8 ± 0.1					75.6 ± 0.1	5.6 ± 0.1
CN6–10	8	12.5 ± 0.1					83.4 ± 0.2	4.1 ± 0.1

In contrast, the Cr–Si-coated CN5–50 sample (Figure 7c) shows a dense and crack-free surface morphology after 50 thermal cycles at 500 °C, indicating excellent mechanical durability. EDX analysis (Spectrum 7, Table 4) identified a much lower oxygen content (18.8 at. %) on the CN5–50 surface compared to that of N5–10 and N5–50 samples, significantly reducing oxidation. It should be noted that the surface of the CN5–10 sample (not shown) displayed a similar morphology to that of the CN5–50 sample, with less oxygen content (12.6 atom %), which shows the protective nature of the Cr–Si coating from the early stages of oxidation. Similarly, the Cr–Si layer kept its uniform coating structure with no visible cracks or spallation when the cyclic oxidation temperature was raised to 600 °C (CN6–10, Figure 7d), confirming the effective oxidation resistance and thermal stability of the coating layer in harsh environments. EDX results (Spectrum 8, Table 4) further support this observation, with only 12.5 at. % oxygen, 83.4 at. % Cr, and 4.1 at. % Si contents, confirming the good chemical stability of the Cr–Si coating even under severe oxidation conditions.

The phase constitutions of uncoated and Cr–Si-coated (Ni,Fe)­TiSb thermoelectric samples subjected to cyclic oxidation are listed in Figure 8. In N5–10, a notable decrease in the NiTiSb peak intensities was observed, and the additional phases of NiSb and TiO_2_ emerged (Figure 8a). After 50 cycles (N5–50), similar peaks as observed for the N5–10 sample were detected but with higher intensities of NiSb and TiO_2_ and weaker for NiTiSb half-Heusler peaks, indicating oxidation-induced phase transformation on the surface after cyclic oxidation. In contrast, the Cr–Si-coated samples (NC, NC5–10, and NC5–50) exhibited an identical phase composition after 10 or 50 cycles, as shown in Figure 8b. The XRD patterns of coated samples revealed consistent NiTiSb crystallographic peaks, reflecting from the substrate due to X-ray penetration, with no detectable peaks corresponding to oxide phases or oxidation products, even after 50 cycles. Strong Cr peaks in all coated samples confirm the structural reliability of the protective coating layer, and Si should be in the solid solution in the Cr phase. It should be noted that the XRD patterns of the N6–10 sample were quite similar to those of the N5–50 sample, with less relative intensity corresponding to TiO_2_ oxide and the NiSb compound.

**Figure 8:**
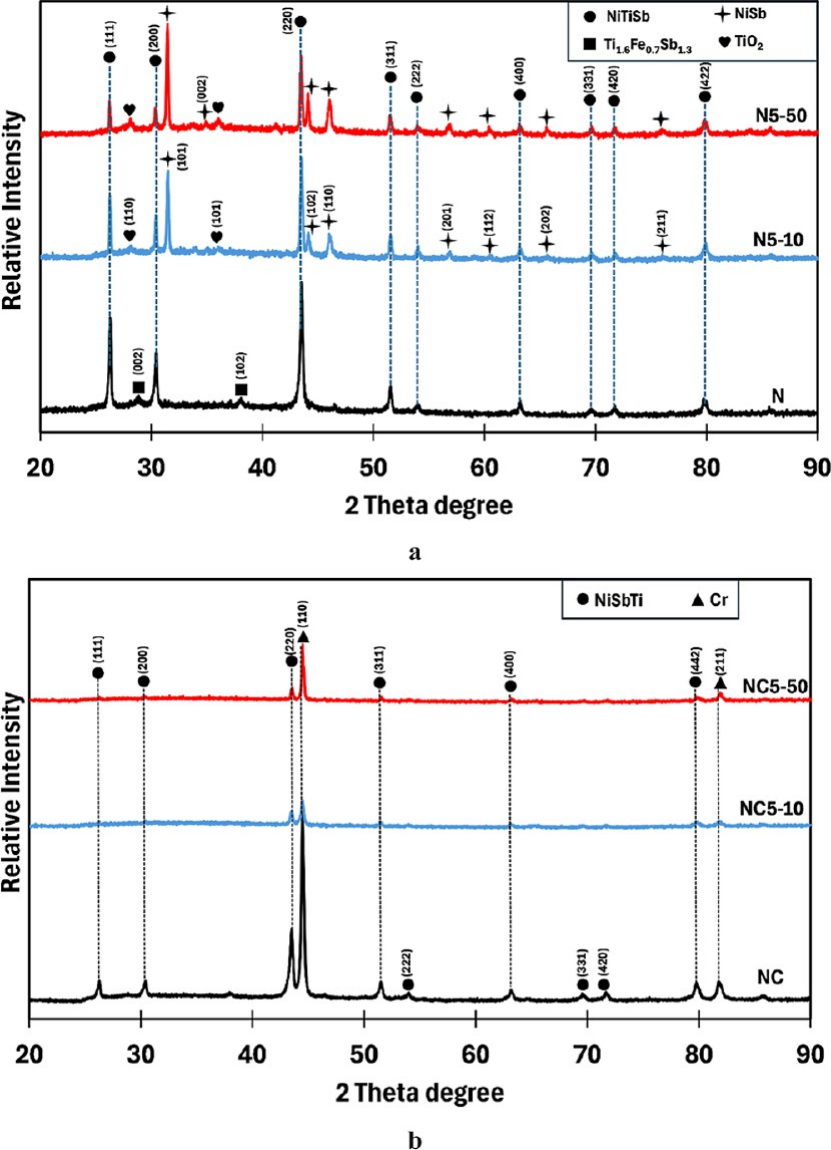
XRD patterns of (a) N, N5–10, and N5–50 samples and (b) NC, NC5–10, and NC5–50 samples.

#### 3.3.3 Cross-Section SEM Observations and Elemental Distributions

The cross-sectional backscattered electron microscope images taken from N5–10, N5–50, CN5–50, and CN6–10 samples are presented in Figure 9. It can be said that the oxidation behavior of uncoated and Cr–Si-coated thermoelectric samples under cyclic oxidation conditions shows a significant differentiation. In the uncoated N5–10 sample (Figure 9a), a multilayered structure (top greyish, middle darker, and underlying bright) formed on the surface, which is different from the matrix, indicating observable oxidation even after a short oxidation period. Once the number of thermal cycles increased to 50, severe deterioration was observed (sample N5–50, Figure 9b), where multilayers observed on the N5–10 sample disappeared; instead, a much complicated, thick, porous, extensively cracked, and delaminated top layer formed, confirming that prolonged cyclic oxidation significantly compromises structural integrity.

**Figure 9:**
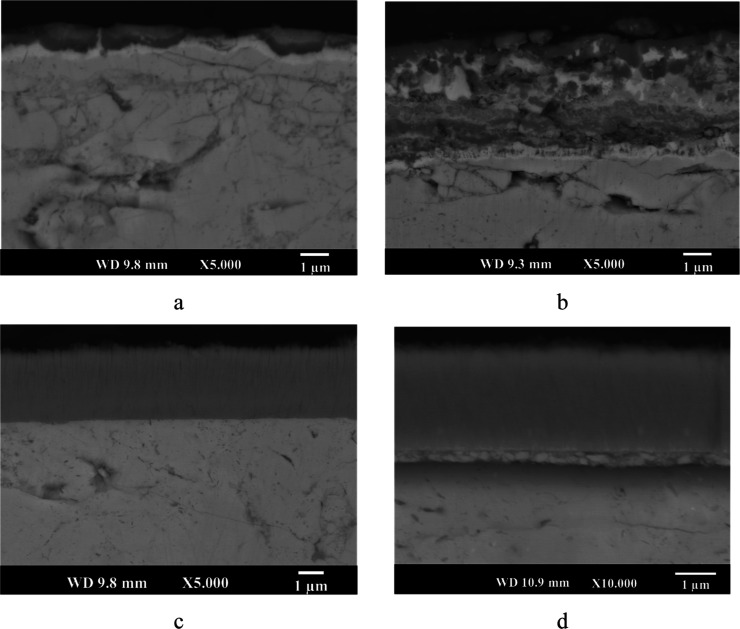
Cross-sectional SEM images of (a) N5–10, (b) N5–50, (c) CN5–50, and (d) CN6–10 (this image was taken at higher magnification to present the interdiffusion layer more clearly).

In contrast, the CN5–50 sample (Figure 9c) exhibits a dense, continuous, and undamaged Cr–Si coating layer that maintains the structural stability of the TE material even after 50 cycles, effectively protecting the substrate material when compared to the N5–50 sample. Similarly, the CN6–10 sample (Figure 9d), imaged at higher magnification, reveals a uniform coating with a thin interdiffusion layer without any defects. These observations confirm that the Cr–Si coating effectively prevented surface oxidation and enhanced the thermal resistance of the substrate TE material.

The typical EDX elemental mappings of the N5–10 and CN6–10 samples are displayed in Figure 10. A significant amount of oxygen and titanium is observed on the uncoated N5–10 sample surface, which is in line with the EDX results presented in Table 4, suggesting the formation of a TiO_2_ oxide layer, as confirmed by the XRD results (see Figure 8a). Below the top region, nickel and antimony are also detected, indicating the presence of NiSb formation. The deep penetration of oxygen into the substrate and the redistribution of the elements near the surface strongly highlight the insufficient chemical stability of the as-produced TE material under short-term cyclic oxidation.

**Figure 10:**
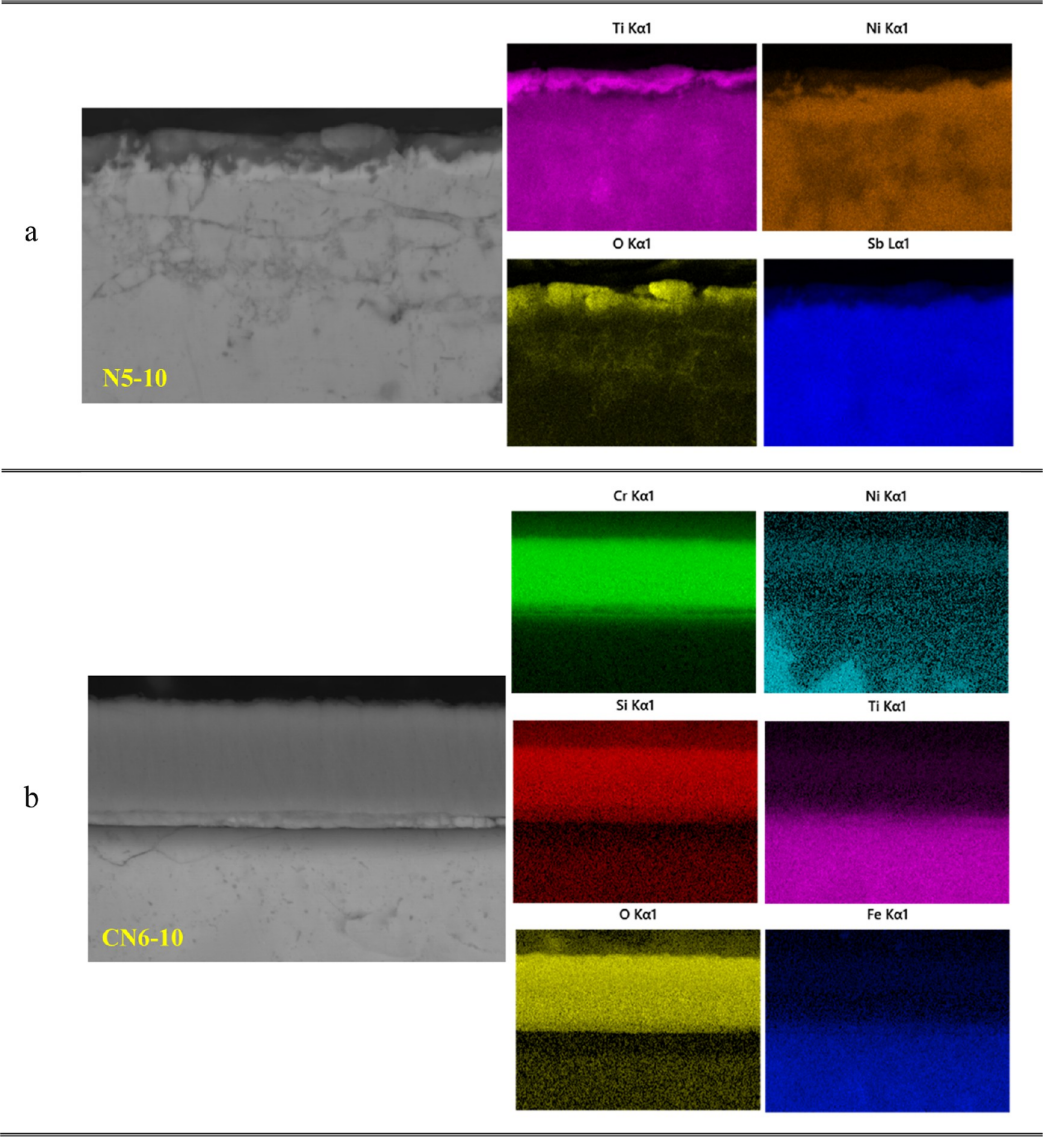
Cross-sectional elemental distribution of (a) N5–10 and (b) CN6–10 samples.

Fortunately, the CN6–10 sample (Figure 10b) reveals uniformly distributed Cr and Si atoms in the coating layer at the surface. The oxygen is also homogeneously present in the coating structure, and its diffusion toward the substrate material is blocked by the attractive Cr–Si layers. It should also be observed that no further oxygen enrichment was observed on the top of the coating layer, meaning that the Cr–Si coating remains chemically stable without forming either Cr_2_O_3_ or SiO_2_ oxides on the coating surface under the high-temperature cyclic oxidation conditions (600 °C-10 h). Beneath the coating, an interphase layer was formed, which consists of Cr and Si elements diffused from the coating layer and Ni, Ti, and Sb elements diffused from the substrate TE material. Although the coating and interdiffusion layers are rich in oxygen content, they significantly reduce oxygen penetration into the TE material compared with the N5–10 sample. Nevertheless, the interface between the interdiffusion layer and the substrate TE material is sharp with no cracks or any other damage, indicating adequate protection against cyclic oxidation.

## 4 Discussion

### 4.1 Comparison of the Static and Cyclic Oxidation Behavior of the (Ni,Fe)­TiSb TE Material

A comparative investigation on the static and cyclic oxidation behavior of the (Ni,Fe)­TiSb thermoelectric material reveals that although both oxidation techniques resulted in surface degradation and morphology alteration, their oxidation mechanisms and degradation characteristics differ depending on the oxidation parameters. The statically oxidized N5 sample exhibited surface cracks and delamination (Figure 2a), similar to the damage observed in the cyclically oxidized N5–10 sample (Figure 7a), suggesting that the identical oxidation mechanism may govern the early stage oxidation-related degradation in both tests, more likely associated with volume expansion of the TiO_2_ oxide layer. XRD patterns (Figures 3a and 8a) confirmed NiSb and TiO_2_ phases in both oxidation methods, supporting this observation. The cross-sectional SEM images of both N5 and N5–10 (Figures 4a and 9a, respectively) revealed that the formed oxide scales were loosely bonded to the surface, which may have facilitated further oxygen diffusion, as confirmed by the EDX analyses (approximately 60–70%, Tables 3 and 4), indicating that both oxidation types rapidly altered the original matrix composition and promoted continued surface degradation. Similar observations were made for the N6–10 sample (not presented due to the similar behavior) when compared to the N5 and N5–10 samples, at which the temperature increased to 600 °C.

It can also be said that a two-layer structure (top TiO_2_ and sub-NiSb) was observed on the N5, N5–10 (see Figures 4a and 9a), and N6–10 (not presented as it is similar to N5–10) samples, which can be attributed to the decomposition of the NiTiSb matrix because of Ti diffusion toward the surface to meet the oxygen, thus forming a TiO_2_ outer layer, as the affinity to oxygen of Ti is greater than that of Ni, Fe, and Sb.^29^ In addition, Ni and Sb remained the same without diffusion below the top TiO_2_ layer and reacted with each other to form a secondary NiSb phase. As the oxidation temperature and duration increased, particularly in the N6 sample, more Ti outward diffusion was observed, leading to formation of thicker top-TiO_2_ and sub-NiSb layers (Figure 4c). It is thought that a similar multilayered structure formed on the N5–50 sample was distinctly deformed due to repetitive thermal cycling that induced internal stresses, severely worsening the mechanical integrity of the TiO_2_ and NiSb layers, resulting in their massive degradation (Figure 9b).

The comparison of oxidation behavior in N5–50 and N6 samples provides insight into the effects of temperature (500/600 °C) and oxidation method (static/cyclic). Even though N6 was oxidized at a higher temperature (600 °C), a greater mechanical degradation was observed in the N5–50 sample surface, as presented in the cross-sectional SEM images (N6 in Figure 4c and N5–50 in Figure 9b), showing massive cracks, delamination, and spallation. The reason for such degradation can be explained by the internal stress increase that occurs during repeated heating and cooling. In contrast, the thick TiO_2_ and NiSb layers on the N6 surface remained, although some spallation was observed on the surfaces (Figure 4c). The primary reason for the progressive mass gain in N5–50 is the cyclic oxidation conditions, which enable continuous oxygen penetration into the fresh matrix after the extensively degraded surface is removed due to oxidation.

These comparisons demonstrate that static oxidation leads to the formation of thermodynamically stable but loosely bonded oxides, resulting in layer delamination and rapid structural transformation. In contrast, cyclic oxidation triggers massive mechanical degradation, causing progressive oxygen diffusion, yet it may still allow for the partial existence of the matrix phase. In both cases, it can be understood that the novel surface engineering approach is essential for protecting this TE material against static and cyclic oxidation, thereby developing long-life TE modules.

### 4.2 Cr–Si Coating against Degradation in both Static and Cyclic Oxidation Modes

The Cr–Si coating provided effective oxidation protection for the (Ni,Fe)­TiSb TE substrate under both static and cyclic oxidation conditions, maintaining its structural and chemical stability. The coating’s integrity, without cracks or spallation at the surface and the interface between the CrSi coating and TE substrate, indicates good adhesion and thermal compatibility with the substrate. Surface morphology of static (CN6, Figure 2c) and cyclic (CN5–50 and CN6–10, Figure 7c,d) samples consistently revealed a dense, uniform, and continuous coating layer, with no observable surface oxide particles. The oxygen content was lower in cyclically oxidized samples, 18.8% for CN5–50 and 12.5% for CN6–10 (Table 4) compared to 26.7% in static CN6 (Table 3), meaning the static oxidation at 600 °C for 50 h was the extreme among the selected experimental parameters.

XRD analyses supported surface morphology observations, showing that neither static nor cyclic exposure resulted in the formation of Cr_2_O_3_ or SiO_2_ phases (Figures 3b and 8b), nor postoxidation substrate products such as TiO_2_ or NiSb, which were detected in uncoated samples (Figures 3a and 8a), indicating the coating’s strong chemical stability and the effective isolation of the substrate from the oxidizing environment, even after thermal cycling. These findings are in agreement with the previously published study,^30^ showing that the Cr_2_O_3_ or SiO_2_ phase can be observed for longer oxidation duration (80 h) at 600 °C or higher temperatures (700 °C/800 °C).

Cross-sectional SEM and EDX mapping provide further insight into the coating’s efficiency. As seen in the CN5 and CN5–50 samples (Figures 4b and 9c), the Cr–Si layer provided a sharp interface, with no cracks, delamination, or substrate degradation. This observation confirms the sufficient adhesion of the Cr–Si layer to the (Ni,Fe)­TiSb substrate, although no direct bonding strength test was conducted in this study. Additionally, this strong interfacial integrity likely contributed to the excellent antioxidant performance observed under both static and cyclic oxidation conditions. For the CN6 and CN6–10 samples (Figures 4d and 9d), an interdiffusion zone formed between the coating and substrate, resulting from the diffusion of elements from the coating and substrate (Figures 5b and 10b). Although this interlayer indicates an ongoing interaction at elevated temperatures, it did not compromise the barrier performance. In addition, oxygen was concentrated within the coating and interdiffusion regions rather than in the substrate, indicating that these zones blocked the diffusion of oxygen to the substrate. It is clear that oxygen is diffused into the coating layer along the grain boundaries and is uniformly distributed within it. As shown in Figure 1, the Cr–Si layer forms with a columnar structure, creating a pathway for oxygen diffusion. Nevertheless, the thermodynamically attractive Cr-rich layer retained the oxygen atoms and protected the substrate TE material against oxidation. It was also thought that such interdiffusion layer formation may reinforce the interface over time by enhancing bonding and reducing thermal mismatch stress during cyclic exposure, although an intrinsic coefficient of thermal expansion mismatch between Cr–Si and the half-Heusler substrate is possible. This may also prevent microcracks or delamination at the coating–substrate interface in any Cr–Si-coated sample after oxidation, even at 600 °C. These observations are consistent with a previously published study on the efficiency of Si-doped Cr-rich coatings in protecting a stainless-steel substrate against oxidation up to 800 °C for a longer duration (80 h).^30^ It can be predicted that this coating can protect HH thermoelectric materials up to 600 °C for longer durations.

The mass gain data further support the effectiveness of the Cr–Si coating in protecting the (Ni,Fe,TiSb) thermoelectric substrate. As confirmed from Figure 6, the CN5–50 and CN6–10 samples exhibited significantly lower mass gain compared to the uncoated N5–50 and N6–10 samples, indicating a considerable reduction in oxygen inward diffusion. The minimal mass gain and the above-mentioned microstructural investigations confirm the thermal barrier characteristic of the Cr–Si coating layer in preventing oxidation at high temperatures.

## 5 Conclusions

Based on the results, the following key conclusions were drawn:

- The as-produced (Ni,Fe)­TiSb thermoelectric material exhibited considerable surface morphology changes after oxidation at 500 and 600 °C. These alterations occurred under both static and cyclic oxidation conditions, leading to disruption of the structured half-Heusler phase near the surface, which may impair carrier mobility and reduce overall thermoelectric performance.
- In uncoated (Ni,Fe)­TiSb samples, severe surface cracking, spallation, and oxide layer formation were observed, particularly under cyclic oxidation. The sample subjected to 50 thermal cycles (N5–50) displayed the most pronounced damage, primarily due to repeated thermal stresses and the deterioration of postoxidation products such as the TiO_2_ oxide scale and the NiSb compound.
- The Cr–Si-coated samples retained surface integrity without structural failure, even after prolonged exposure to elevated temperatures. The coating effectively suppressed oxygen diffusion toward the substrate due to the strong affinity of Cr and Si atoms for oxygen. Oxygen entrapment within the coating layer contributed to the stabilization of the substrate chemical composition, thereby preserving the integrity of the TE material.
- Although interdiffusion layers were observed in the statically oxidized CN6 and cyclically oxidized CN6–10 samples, both the Cr–Si coating and the interfacial zones served as effective barriers against oxygen diffusion. Oxygen ingress into the substrate was successfully inhibited, thus protecting the TE material from oxidation.

These findings clearly demonstrate that Cr–Si coatings offer effective protection for N-type (Ni,Fe)­TiSb half-Heusler TE materials under both static and cyclic oxidations up to 600 °C.
